# Predictive and prognostic value of excision repair cross-complementing group 1 in patients with advanced gastric cancer

**DOI:** 10.1038/s44276-024-00046-w

**Published:** 2024-03-05

**Authors:** Yasuhide Yamada, Kengo Nagashima, Mizutomo Azuma, Mitsuko Masutani, Hitoshi Ichikawa, Satoru Iwasa, Naoki Takahashi, Hidekazu Hirano, Keisuke Kanato, Nozomu Machida, Takahiro Kinoshita, Hiroaki Hata, Hisato Kawakami, Daisuke Takahari, Narikazu Boku, Yukinori Kurokawa, Masanori Terashima, Takaki Yoshikawa, Shigeki Sekine, Nobuyoshi Hiraoka

**Affiliations:** 1 Department of Medical Research, National Center for Global Health and Medicine, Tokyo, 162-8655 Japan; 2 Biostatistics Unit, Clinical and Translational Research Center, Keio University Hospital, Tokyo, 160-8582 Japan; 3 Department of Gastroenterology, Kitasato University Hospital, Sagamihara, 252-0329 Japan; 4 Molecular & Genomic Biomedicine, Center for Bioinformatics and Molecular Medicine, Nagasaki University Graduate School, Nagasaki, 852-8521 Japan; 5 Department of Clinical Genomics, National Cancer Center Research Institute, Tokyo, 104-0045 Japan; 6 Department of Gastrointestinal Medical Oncology, National Cancer Center Hospital, Tokyo, 104-0045 Japan; 7 Department of Gastroenterology, Saitama Cancer Center, Saitama, 362-0806 Japan; 8 JCOG Data Center/ Operations Office, National Cancer Center, Tokyo, 104-0045 Japan; 9 Department of Gastroenterology, Kanagawa Cancer Center, Yokohama, 241-8515 Japan; 10 Department Gastric Surgery, National Cancer Center Hospital East, Chiba, 241-8515 Japan; 11 Department of Surgery, Kyoto Medical Center, Kyoto, 612-0861 Japan; 12 Department of Medical Oncology, Kindai University Faculty of Medicine, Osaka, 577-8502 Japan; 13 Department of Gastroenterological Chemotherapy, Cancer Institute Hospital of JFCR, Tokyo, 135-8550 Japan; 14 Department of Gastroenterological Surgery, Osaka University Graduate School of Medicine, Osaka, 565-0871 Japan; 15 Division of Gastric Surgery, Shizuoka Cancer Center, Shizuoka, 411-8777 Japan; 16 Department of Gastric Surgery, National Cancer Center Hospital, Tokyo, 104-0045 Japan; 17 Department of Pathology, National Cancer Center Hospital, Tokyo, 104-0045 Japan

## Abstract

**Background:**

To define the optimal chemotherapy regimen for each patient we therefore used tissue from patients to identify molecular prognostic or predictive biomarkers.

**Methods:**

Endoscopic biopsy specimens from primary lesions and surgical specimens on a phase III trial in patients with unresectable advanced or recurrent gastric cancer treated with docetaxel with cisplatin plus S-1 (DCS) or cisplatin plus S-1 (CS), were collected. We measured the mRNA expression of *ERCC1* and analyzed SNPs in *GSTP1* and *ERCC1*.

**Results:**

Low *ERCC1* expression was associated with favorable prognosis for overall survival, OS by multivariable analysis (*P* = 0.001). There were significant interactions between the two treatment arms of DCS and CS, and *ERCC1* mRNA expression. In patients with low *ERCC1* expression of a favorable prognosis, DCS therapy was inferior to CS (*P* = 0.046). In addition to *GSTP1* rs1695 (HR 1.728), *ERCC1* rs3212980, *ERCC1* rs2298881, *ERCC1* rs3212964 with high expression of *ERCC1* mRNA were associated with significantly worse prognosis with regard to OS.

**Conclusions:**

*ERCC1* mRNA is an independent prognostic factor and predictive marker that can be used to guide the addition of docetaxel. The SNPs of *ERCC1* and *GSTP1* could be also prognostic or predictive factors.

## Introduction

Fluoropyrimidine and platinum-based combination therapies are the most commonly used and acceptable first-line therapies for patients with HER-2 negative gastric cancer worldwide [1]. The V325 study demonstrated the superiority of triplet chemotherapy using docetaxel plus cisplatin and 5-fluorouracil (5-FU) over doublet chemotherapy with cisplatin and 5-FU for patients with advanced gastric cancer [2]. This triplet regimen has not been accepted globally as a standard palliative treatment because it elicits severe neutropenia and confers a small survival advantage. JCOG 1013 trial showed that the triplet therapy with docetaxel added to cisplatin and S-1 (DCS) did not prolong overall survival (OS) and progression-free survival (PFS) in patients with unresectable advanced or recurrent gastric cancer compared with the doublet of cisplatin and S-1 (CS) [3]. Poor performance status (PS), peritoneal metastasis, liver metastasis, histological type, and disease status (unresectable advanced or recurrent) are established clinical prognostic factors for metastatic gastric cancer [4, 5]. On the other hand, perioperative triplet therapy with docetaxel, fluoropyrimidine, and oxaliplatin showed survival benefit for patients with locally advanced resectable gastric cancer [6, 7]. These mixed results show that a better understanding of biological predictive or prognostic markers of conventional cytotoxic agents is required. Armed with this knowledge, physicians then can give patients the optimal drugs to prolong their survival and improve their quality of life. This is especially important for the use of cytotoxic drugs, which are not always effective in every patient and often cause severe adverse events.

Excision repair cross-complementation group 1 (ERCC1) is an important component of the nucleotide excision repair pathway, which repairs DNA intra-strand, inter-strand, and DNA-protein crosslinks caused by cisplatin. DNA repair systems allow cells to overcome the DNA damage induced by chemotherapy [8]. In the JCOG9912 trial, low *ERCC1* mRNA expression was a significant independent favorable prognostic factor in patients with metastatic gastric cancer who received first-line chemotherapy with 5-FU monotherapy, S-1 monotherapy, or cisplatin plus irinotecan [9]. Low mRNA levels of *ERCC1* in primary gastric cancer have been associated with a higher overall response rate and longer survival following cisplatin treatment [9–14]. The expression of *ERCC1* mRNA was suggested as a predictive and prognostic marker in resectable gastric cancer patients receiving chemotherapy. Providing complementary roles to *ERCC1*, *X-ray repair cross-complementing group* (*XRCC1*) is critical mediator of base excision repair and single-strand break repair [15, 16].

Single nucleotide polymorphisms (SNPs) *ERCC1* rs3212986, rs2298881, rs11615, *XRCC1* rs25487, and rs1695 in *glutathione S-transferase pi 1* (*GSTP1*; an enzyme that is involved in cytosolic platinum detoxification [16, 17]), have been suggested as prognostic markers in preclinical studies [18–22]. The *ERCC1* genotypes had no significant association with OS in patients who received perioperative therapy with epirubicin, cisplatin, and 5-FU (ECF) in the MAGIC trial [23]. However, patients with a *TYMS* 2 R/2 R genotype derived a larger benefit from perioperative ECF than patients with *TYMS* 3 R genotypes [23]. Although low ERCC1 protein expression may be a better prognostic marker, the lack of adequate commercially available antibodies to detect the active ERCC1 subtype has limited the interpretation of immunohistochemical studies [24–26]. Therefore, we designed the current study to identify differences in survival and tumor regression after CS or DCS therapy. By taking a multi-omics approach, our aim was to quantify the real-world utility of these candidate molecular markers in clinical practice.

## Materials and methods

Patients were randomly assigned (1:1) to receive DCS (docetaxel 40 mg/m^2^ and cisplatin 60 mg/m^2^ on day 1 intravenously, and S-1 40–60 mg twice a day orally for 2 weeks, every 4 weeks) or CS (cisplatin 60 mg/m^2^ intravenously on day 8, and S-1 40–60 mg orally twice a day for 3 weeks, every 5 weeks) in the JCOG1013. Written informed consent to be enrolled in JCOG1013 was obtained before registration and the opportunity to refuse to provide tumor samples was provided through web sites of the National Cancer Center and the Japan Clinical Oncology Group (JCOG) according to the Japanese Ethical Guidelines for Medical and Biological Research Involving Human Subjects. The protocol of this translational study was approved by the institutional review board of the National Center for Global Health and Medicine and each participating hospital and complied according to the criteria of REMARK (reporting recommendations for tumor marker prognostic studies [27].

For the analysis, 5 × 10-μm sections or 10 × 4- or 5-μm sections were prepared from formalin-fixed paraffin embedded tumor tissues (FFPE). The tumor cells on the sections of interest were selectively isolated by macrodissection. *ERCC1* and *TYMS* and an internal reference gene (β-actin) were quantified with a fluorescence-based real-time detection method (LightCycler96 System and FastStart essential DNA Probes Master, Roche Diagnostics, Rotkreuz, Switzerland), both OS and PFS in the patients with lower *ERCC1* mRNA and lower *TYMS* mRNA expression were better than those in the higher *ERCC1* and higher *TYMS* expression in our previous study [9]. The primers and probes used have been described previously [13]. Gene expression values (relative mRNA levels) are expressed as quantification cycle (Cq) ratios (differences between Cq values) between the genes of *ERCC1* or *TYMS* and an internal reference gene (β-actin) [28, 29].

The NCC Oncopanel test is a hybridization capture-based NGS assay designed to examine mutations, amplifications, and homozygous deletions of the entire coding region of 123 genes of clinical or preclinical relevance, along with rearrangements of 13 oncogenes included in the panel [30]. We modified the NCC Oncopanel for pharmacogenetic analysis to examine 66 SNPs in *DPYD*, *VEGFA*, *ABCB1*, *PRKDC*, *MGMT, GSTP1*, *ACRV*, *TYMS*, *XRCC1*, *POLR1G*, and *ERCC1*. We paid particular attention to the genetic changes of *XRCC1* and *GSTP1* that have already been reported to affect the effect of cisplatin as well as *ERCC1*.

Immunohistochemical staining of ERCC1 was performed using antibody 9D11 [31] and an Autostainer Link 48 device (Agilent, Santa Clara, CA). The evaluation area was limited to the region where gastric cancer cells were present in the total tissue of biopsy specimens, and in approximately three locations identified by low magnification (objective lens x4) of the surgical resection samples. The staining intensity was graded on a scale of 0–3. The expression of ERCC1 protein in cancer cells was normalized to the average ERCC1 nuclear staining intensity in intraregional vascular endothelial cells, which was set at 2. Thus, cancer cells expressed similar levels if their average staining intensity was 2, stronger expression if the value was 3, weaker if the value was 1, and were considered negative with a staining intensity of 0. The strongest intensity of ERCC1 expression of cancer cells in the region was measured.

### Statistical analysis

The gene expression levels of *ERCC1* and *TYMS* were categorized into low and high groups by the median or an optimal cutoff value based on a SNP analysis to assess the associations between gene expression levels and OS, and PFS, and response rate. Categorical data were compared using Fisher’s exact test. Survival function was estimated with the Kaplan–Meier method, and differences between survival functions were compared with the log-rank test. Hazard ratios (HRs) with 95% confidence intervals (CIs) based on a Cox proportional hazards model were used to provide quantitative summaries of the gene expression data. Variables for the multivariable analysis included the genes with expression levels (high or low) that showed associations in the univariable analyses in this study, as well as the patient’s background, such as Eastern Cooperative Oncology Group (ECOG) PS, age, sex, number of metastatic sites, previous gastrectomy, presence or absence of target lesions according to RECIST version 1.0, histological classification (differentiated/undifferentiated) [32], and presence or absence of peritoneal metastasis. All reported *P*-values are two-sided, and the level of statistical significance was set at *P* < 0.05. All analyses were performed using R version 4.2.3 (R Foundation for Statistical Computing, Vienna, Austria) and SAS version 9.4 (SAS Institute Inc., Cary, NC).

## Results

### Relationship between *ERCC1* and *TYMS* expression and survival

**Fig. 1:**
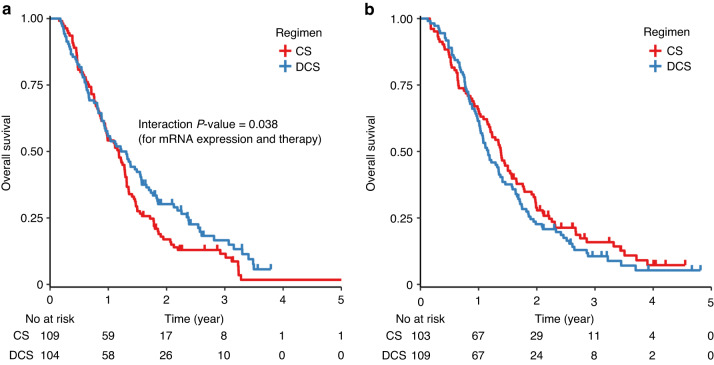
Overall survival (OS) stratified by *ERCC1* mRNA expression and treatment arms. **a** *ERCC1* mRNA expression, median < (*N* = 213), (**b**) ERCC1 mRNA expression, ≤ median (*N* = 212). Patients treated with DCS had longer OS than those with CS in high *ERCC1* mRNA expression (**a**) but low *ERCC1* (**b**). CS cisplatin plus S-1, DCS docetaxel with cisplatin plus S-1.

Tissue samples for this study were collected from 523 endoscopic biopsy specimens and 136 surgical specimens taken before the treatment of 741 randomized patients in JCOG1013 (Supplementary Fig. 1). The baseline characteristics were equally distributed among the subsets for *ERCC1* and *TYMS*. A univariable analysis of the whole study population showed that both OS (HR, 0.861; 95% CI: 0.703–1.054; *P* = 0.147) and PFS (HR, 0.882; 95% CI: 0.726–1.071; *P* = 0.205) in the low *ERCC1* mRNA groups were generally better than those in the high *ERCC1* mRNA groups. There were significant interactions between the two treatment arms of DCS and CS, and *ERCC1* mRNA expression (Table 1). High *ERCC1* mRNA expression was significantly associated with worse prognosis, and DCS was inferior to CS in patients with low *ERCC1* mRNA expression who had a better prognosis (Fig. 1). The response rates of CS and DCS were similar: 43% (47/109) and 36% (37/104) in the *ERCC1*-mRNA high group, and 29% (30/103) and 37% (40/109) in the *ERCC1*-mRNA low group. There were no significant differences in OS or PFS according to the expression of *TYMS*. Multivariable analyses for survival with *ERCC1* mRNA expression and clinical characteristics showed that independent prognostic factors were *ERCC1* mRNA and ECOG PS for OS, and *ERCC1* mRNA and peritoneal metastasis for PFS (Table 2).

**Table 1:** Interaction between treatment arms and expression of *ERCC1* and *TYMS* mRNA with regards to survival.

	OS					PFS				
		Number of patients	HR	95%CI	*P*-value		Number of patients	HR	95%CI	*P*-value
*ERCC1*										
mRNA expression	Median <	213	-	-		Median <	213	-		
	≤ Median	212	0.70	0.53, 0.94	0.017	≤ Median	212	0.75	0.57, 0.99	0.041
Treatment	CS	212	-	-		CS	212	-		
	DCS	213	0.78	0.58, 1.04	0.088	DCS	213	0.85	0.65, 1.12	0.241
Interaction mRNA expression and therapy	425					425				
Median < and DCS		109	1.51	1.01, 2.27	0.046		109	1.42	0.96, 2.10	0.079
*TYMS*										
mRNA expression	Median <	224	-	-		Median <	224	-		
	≤ Median	224	0.82	0.62, 1.08	0.153	≤ Median	224	0.85	0.65, 1.12	0.245
Treatment	CS	224	-	-		CS	224	-		
	DCS	224	0.88	0.67, 1.17	0.389	DCS	224	0.99	0.75, 1.29	0.926
Interaction mRNA expression and therapy	448					448				
Median < and DCS		118	1.15	0.78, 1.72	0.478		118	1.03	0.70, 1.50	0.890

*OS* overall survival, *PFS* progression-free survival, *HR* hazard ratio, *CI* confidence interval, *Cq, CS* cisplatin and S-1, *DCS* docetaxel, cisplatin, and S-1.

**Table. 2:** Multivariate analysis for overall survival and progression-free survival.

	OS			PFS		
Characteristic	HR	95% CI	*P*	HR	95% CI	*P*
*ERCC1* mRNA						
High	—	—		—	—	
Low	0.32	0.16, 0.63	0.001	0.34	0.18, 0.63	<0.001
ECOG PS						
0	—	—		—	—	
1	1.89	1.20, 2.98	0.006	1.29	0.85, 1.98	0.236
Age				—	—	
≤65	—	—		—	—	
>65	1.44	0.92, 2.26	0.114	1.34	0.87, 2.05	0.179
Sex						
Male	—	—		—	—	
Female	1.13	0.69, 1.84	0.631	0.98	0.61, 1.55	0.920
No. of metastatic sites						
0–1	—	—		—	—	
2 ≤	1.50	0.91, 2.46	0.110	1.46	0.92, 2.31	0.107
Previous gastrectomy						
No	—	—		—	—	
Yes	1.01	0.60, 1.71	0.975	1.38	0.83, 2.29	0.215
Measurable lesion						
No	—	—				
Yes	0.92	0.52, 1.61	0.761	1.62	0.94, 2.77	0.080
Histology						
Differentiated adenocarcinoma	—	—		—	—	
Undifferentiated adenocarcinoma	0.94	0.57, 1.54	0.805	0.93	0.59, 1.46	0.738
Peritoneal metastasis						
No	—	—		—	—	
Yes	1.72	0.93, 3.20	0.086	2.15	1.22, 3.80	0.008

*OS* overall survival, *PFS* progression-free survival, *HR* Hazard Ratio, *CI* confidence interval.

### Relationship between *ERCC1* mRNA and protein expression

There was no statistically significant correlation between *ERCC1* Cq ratio and protein expression. The protein staining intensities (scale 0–3) in the *ERCC1* mRNA*-*high group were 3 in 32/142 (23%), 2 in 84/142 (46%), 1 in 20/142 (38%), and 0 in 6/142 (4%). Staining intensities in the *ERCC1-*mRNA low group were 3 in 51/196 (26%), 2 in 98/196 (54%), 1 in 33/196 (17%), and 0 in 14/196 (7%). ERCC1 expression had no predictive and prognostic significance with regard to OS, PFS or tumor shrinkage.

### *ERCC1*, *XRCC1*, and *GSTP1* SNPs as prognostic factors

Genomic analysis using the NCC Oncopanel was performed on 111 surgical specimens and 13 endoscopic biopsy samples; most patients were postoperative recurrent cases. DCS was superior to CS for patients with recurrent gastric cancer after gastrectomy in terms of OS (21.9 months [95%CI, 17.9–26.3] vs 15.9 months [12.9–19.0], HR = 0.64 [0.45–0.90], *P* = 0.0095), but not superior in patients with unresectable advanced gastric cancer (Supplementary Fig. 2 MST, 13.0 months [11.9–14.3] vs 15.0 months [14.1–16.1], HR = 1.14 [0.963–1.36], *P* = 0.127). There were significant differences between the baseline patient characteristics of unresectable advanced and recurrent gastric cancer regarding PS 1 (37% vs. 25%), only one metastatic site (38% vs. 66%), liver metastasis (31% vs. 21%), and bone metastasis (5% vs. 10%). Thus, patients with recurrent gastric cancer had more favorable prognostic factors than those with unresectable advanced gastric cancer. Among the 124 patients for whom data were available, DCS was superior to CS (*P* < 0.01). There were no differences in the distribution of *ERCC1* mRNA expression between patients with unresectable advanced and those with recurrent gastric cancer. The prognostic values of *ERCC1*, *XRCC1*, and *GSTP1* in patients treated with DCS or CS are shown in Table 3. *ERCC1* rs3212964 (HR 1.533), *ERCC1* rs2298881 (HR 1.525), and *GSTP1* rs1695 (HR 2.336) were significant prognostic factors with regard to PFS. The *ERCC1* rs3212980 TT, rs3212964 TT, rs11615 AA, rs3212948 GG, and rs2298881 AA alleles tended to have higher mean values of *ERCC1* mRNA expression when compared with the reference alleles. Other remarkable HRs of DCS vs CS in terms of OS were 0.259 in *DPYD* rs2297595 TC (vs 0.514 in TT), 1.777 in *ABCB1* rs7787082 AA (vs 0.435 in GA and 0.426 in GG), 0.237 in *XRCC2* rs1799782 AA (vs 0.499 GA and 0.678 in GG) (Supplementary Table 2).

**Table 3:** *ERCC1* SNPs and mRNA as prognostic factors.

Gene	rsID	Allele	*N*	mRNA, Cq ratio		OS			PFS		
				Mean	SD	HR	95%CI		HR	95%CI	
*ERCC1*	3212980	GG/TG	42	0.142	0.342	Ref.			Ref.		
		TT	82	0.040	0.142	1.331	0.880	2.014	1.345	0.913	1.981
	3212964	CC/CT	93	0.094	0.268	Ref.			Ref.		
		TT	31	0.017	0.020	1.259	0.809	1.958	1.533	1.007	2.333
	11615	AG/GG	113	0.086	0.254	Ref.			Ref.		
		AA	11	0.020	0.015	1.175	0.609	2.266	0.777	0.406	1.489
	3212948	CC/GC	113	0.086	0.254	Ref.			Ref.		
		GG	11	0.020	0.015	1.175	0.609	2.266	0.777	0.406	1.489
	2298881	CA/CC	95	0.096	0.270	Ref.			Ref.		
		AA	29	0.016	0.019	1.330	0.845	2.092	1.525	0.994	2.340
*XRCC1*	rs25487	TC/CC	117	-	-	Ref.			Ref.		
		TT	7	-	-	1.024	0.448	2.341	1.671	0.773	3.611
	rs1799782	GA/GG	109	-	-	Ref.			Ref.		
		AA	15	-	-	0.762	0.384	1.512	0.956	0.526	1.740
*GSTP1*	rs1695	AG/AA	118	-	-	Ref.			Ref.		
		GG	6	-	-	1.728	0.751	3.978	2.336	1.016	5.368

High value of Cq ratio, threshold cycle *ERCC1*/β-actin, means low *ERCC1* expression.

*OS* overall survival, *PFS* progression-free survival, *RR* response rate, *SD* standard deviation, *P* *p*-value, *HR* hazard ratio, *Ref.* reference, *CI* confidence interval.

### *ERCC1*, *XRCC1*, and *GSTP1* SNPs as predictive factors

*ERCC1*, *XRCC1*, *GSTP1* SNPs were predictive of the benefits of DCS or CS in terms of OS and PFS (Table 4). When compared with each reference allele, the *ERCC1* rs3212980 TT, rs3212964 TT, rs11615 AA, rs3212948 GG and rs2298881 AA alleles tended to have larger HR for both OS and PFS in the DCS-treated patients versus those treated with CS. Patients with *GSTP1* GG who were treated with DCS had a shorter OS. The response rates in the CS and DCS groups were 20% vs. 27% in *ERCC1* rs3212980 TT, 6% vs. 24% in rs3212964 TT, 0% vs. 29% in rs11615 AA, 0% vs. 29% in rs3212948 GG, and 6% vs. 25% in rs2298881 AA.

**Table 4:** SNPs of *ERCC1*, *XRCC1*, and *GSTP1* as predictive factors.

Gene	rsID	Allele	*N*	Comparison	OS			PFS		
					HR	95%CI		HR	95%CI	
*ERCC1*	3212980	GG/TG	42	DCS vs CS	0.255	0.113	0.578	0.429	0.220	0.838
		TT	82		0.704	0.437	1.132	0.689	0.439	1.082
	3212964	CC/CT	93		0.469	0.295	0.747	0.562	0.365	0.865
		TT	31		0.812	0.368	1.790	0.667	0.314	1.417
	11615	AG/GG	113		0.493	0.325	0.746	0.519	0.352	0.767
		AA	11		0.807	0.191	3.416	1.251	0.309	5.067
	3212948	CC/GC	113		0.493	0.325	0.746	0.519	0.352	0.767
		GG	11		0.807	0.191	3.416	1.251	0.309	5.067
	2298881	CA/CC	95		0.501	0.318	0.789	0.557	0.363	0.854
		AA	29		0.691	0.302	1.580	0.685	0.313	1.503
*XRCC1*	25487	TC/CC	117	DCS vs CS	0.517	0.346	0.775	0.561	0.383	0.821
		TT	7		0.572	0.060	5.437	1.724	0.284	10.465
	1799782	GA/GG	109		0.576	0.382	0.869	0.599	0.405	0.888
		AA	15		0.237	0.048	1.162	0.325	0.095	1.116
*GSTP1*	1695	AG/AA	118	DCS vs CS	0.532	0.355	0.796	0.588	0.402	0.860
		GG	6		4.472	0.279	71.807	0.592	0.062	5.654

*OS* overall survival, *PFS* progression-free survival, *RR* response rate, *PD* Progressive disease, *SD* standard deviation, *Est.* estimated, *Ref.* reference, *CI* confidence interval.

### Intra-tumoral gene mutation and outcomes

**Fig. 2:**
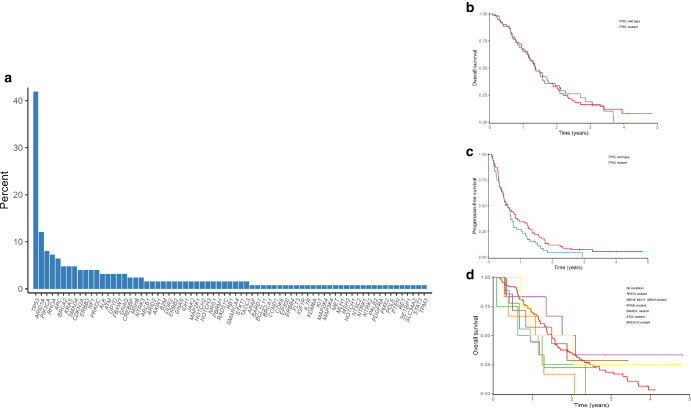
Incidence of somatic mutation and survival in metastatic gastric cancer. **a** *TP53* mutation was most commonly observed mutation in gastric cancer. **b** TP53 status had no impact on OS following treatment with either CS or DCS (HR, 1.02; 95% confidence interval, 0.69–1.51). **c** Patients with wild type *TP53* had better PFS than those with *TP53* mutant (HR, 1.33; 95% confidence interval, 0.91–1.93). **d** OS of patients segregated based on mutations of representative genes.

*TP53* mutation was observed in 43% of cases, *ARID1A* in 12%, *PIK3CA* in 8.1%, *RHOA* in 7.3%, *APC* in 6.5%, *BRCA2* in 4.8%, *KRAS* in 4.8%, *SMAD4* in 4.8%, *MSH6* in 2.4%, *MLH1* in 0.8%, and *MSH2* in 0.8% (Fig. 2). There was no difference of OS between mutant and wild-type of *TP53* (HR 1.02; 95%CI: 0.69 to 1.51; *P* = 0.91). There was a tendency of poorer OS in patients with *RHOA*, *SMAD4*, or microsatellite instability- high (*MSH6*/*MLH1*/*MSH2*) genetic alterations.

## Discussion

DNA repair capacity is a major determinant of cisplatin resistance, with ERCC1 protein playing an essential role in nucleotide excision repair. Here, we found that patients with high *ERCC1* expression had a poorer overall survival than those with low *ERCC1* expression. The DCS treatment regimen was inferior to CS in patients with low *ERCC1* expression and a favorable prognosis. Therefore, the triplet therapy is not required in this patient subset. Recurrent gastric cancer patients with *ERCC1* rs3212964, *ERCC1* rs2298881, or *GSTP1* rs1695 SNPs had higher *ERCC1* expression and had a worse OS. Moreover, DCS was inferior to CS in terms of PFS if patients had the *ERCC1* rs11615 AA or *ERCC1* rs3212948 GG SNPs. DCS was also inferior to CS in terms of OS in patients with the *XRCC1* rs25487 TT or *GSTP1* rs1695 GG SNPs. Our data confirmed that *ERCC1* expression, as well as specific SNPs in *ERCC1*, *XRCC1*, and *GSTP1*, are significant prognostic indicators that could guide the choice between DCS or CS treatment regimens. There were no significant differences in the impact of *ERCC1* mRNA expression on prognosis between *TP53* mutant and wild-type (*P* = 0.5, Pearson’s Chi-squared test).

Our previous ancillary investigation of another randomized controlled trial, JCOG9912, showed that low *ERCC1* expression was a significant independent favorable prognostic factor in patients with advanced gastric cancer who were also receiving first-line chemotherapy. The baseline patient characteristics were different between JCOG9912 and the current JCOG1013 trial. About 50% of analyzed patients in JCOG1013 had peritoneal metastasis compared with 27% in JCOG9912. The frequency of liver metastasis was 30% in this study but 48% in JCOG9912. Although the treatment regimens were different (CS and DCS were used in JCOG1013 whereas 5-FU monotherapy, S-1 monotherapy, or cisplatin plus irinotecan combination therapy were used in JCOG9912) the prognostic effect of *ERCC1* expression was still evident in both cases. The *ERCC1* rs3212964 and *ERCC1* rs2298881 SNPs were found in patients with higher *ERCC1* expression, which explains why they were associated with a poorer prognosis. Because genotyping from FFPE breast cancer specimens was significantly concordant with genotyping from germline DNA, the effects of cytotoxic chemotherapy and its impact on survival can be predicted by DNA analysis of blood or buccal mucosa [33].

Since high *ERCC1* expression is an indicator of poor prognosis, it has been a challenge to show the superiority of alternative combination therapies without platinum with regard to survival [14, 34]. From our results, some patients with low *ERCC1* expression have a good prognosis, and this is compromised if they are given the more toxic triplet therapy. Hence, the administration of DCS to patients with an otherwise favorable prognosis, particularly those who are eligible for curative resection. should be avoided. Commercially available methods to evaluate *ERCC1* mRNA expression status are warranted, as they will guide the choice of triplet DCS or doublet CS, which in turn will reduce the incidence of toxicity-related death and will improve patients’ quality of life. S-1 was effective in *ERCC1*-high patients with resectable stage II or III gastric cancer after surgery in the adjuvant setting. However, S-1 monotherapy did not impart a statistically significant survival benefit in *ERCC1*-low patients in the ACTS-GC trial [35]. Therefore, *ERCC1* mRNA expression could be predictive marker in the adjuvant setting. Thus, ERCC1 is not only related to the resistance of cisplatin but other chemotherapeutic agents. The results of our present study, which show that DCS is more effective than CS in *ERCC1*-high patients, are consistent with these previously published data.

The *ERCC1* gene generates four isoforms designated 201, 202, 203, and 204 by alternative splicing. Currently, available antibodies such as 8F1 cannot discriminate between these isoforms and thus cannot guide therapeutic decision-making regarding cisplatin combined therapy in patients with non-small-cell lung cancer; this requires specific detection of the unique functional ERCC1-202 isoform [26]. ERCC1 expression was analyzed by western blot in seventeen human gastric cancer cell lines, and all were found to express either 201, 202, and/or 203, but not 204 [31]. Although domain-specific functions that are clinically relevant to the 202 isoform of ERCC1 have been identified, there is a lack of structure-function data for the other isoforms with regard to cisplatin resistance. Considering this, we suggest that antibodies capable of detecting not only 202 but also other major ERCC1 isoforms may be useful for evaluation of cisplatin sensitivity. Therefore, we used 9D11, a novel antibody that recognizes ERCC1 isoforms 201, 202, and 203 [31]. We did not, however, identify a significant prognostic impact of ERCC1 protein expression when using this 9D11 antibody. Evaluating ERCC1 protein expression levels using anti-ERCC1 antibodies is not useful for predicting the prognosis of patients receiving cisplatin combination therapy. In conclusion, we believe that this is the first study to evaluate the prognostic and predictive value of *ERCC1* gene alteration, *ERCC1* mRNA expression, and *GSTP1* polymorphism in patients with unresectable or recurrent gastric cancer. We demonstrate that genomic and transcriptomic analyses can guide the selection of cytotoxic chemotherapy and recommend that gene sequencing is performed before selecting patients for specific treatment regimens.

## Supplementary information

Supplementary Fig.

Supplementary Fig.

Supplementary table

## Data Availability

The row data of SNPs about *DPYD*, *VEGFA*, *ABCB1*, *PRKDC*, *MGMT*, *GSTP1*, *ACRV*, *TYMS*, *XRCC1*, *POLR1G*, and *ERCC1* were shown in Supplementary Tables 1 and 2.
