# Supplementary material for: Predictive and prognostic value of excision repair cross-complementing group 1 in patients with advanced gastric cancer
Source: BJC Rep. 2024 Mar 5;2:18. doi: 10.1038/s44276-024-00046-w (PMC11523942; doi:10.1038/s44276-024-00046-w)
Supplement: Supplementary file 1 — Supplementary Fig. [file 44276_2024_46_MOESM1_ESM.docx]

Supplementary Figure 1. Samples for analyses

ERCC1, excision repair cross-complementation group 1; TYMS, thymidylate synthase

Supplementary Figure 2. Overall survival in patients with unresectable advanced or recurrent metastatic gastric cancer

The docetaxel with cisplatin plus S-1 therapy was significantly superior to cisplatin plus S-1 therapy for patients with recurrent gastric cancer after gastrectomy (b). However, it was not superior in patients with unresectable advanced gastric cancer (a).
