# Supplementary material for: Predictive and prognostic value of excision repair cross-complementing group 1 in patients with advanced gastric cancer
Source: BJC Rep. 2024 Mar 5;2:18. doi: 10.1038/s44276-024-00046-w (PMC11523942; doi:10.1038/s44276-024-00046-w)
Supplement: Supplementary file 2 — Supplementary Fig. [file 44276_2024_46_MOESM2_ESM.pptx]

## Slide 1
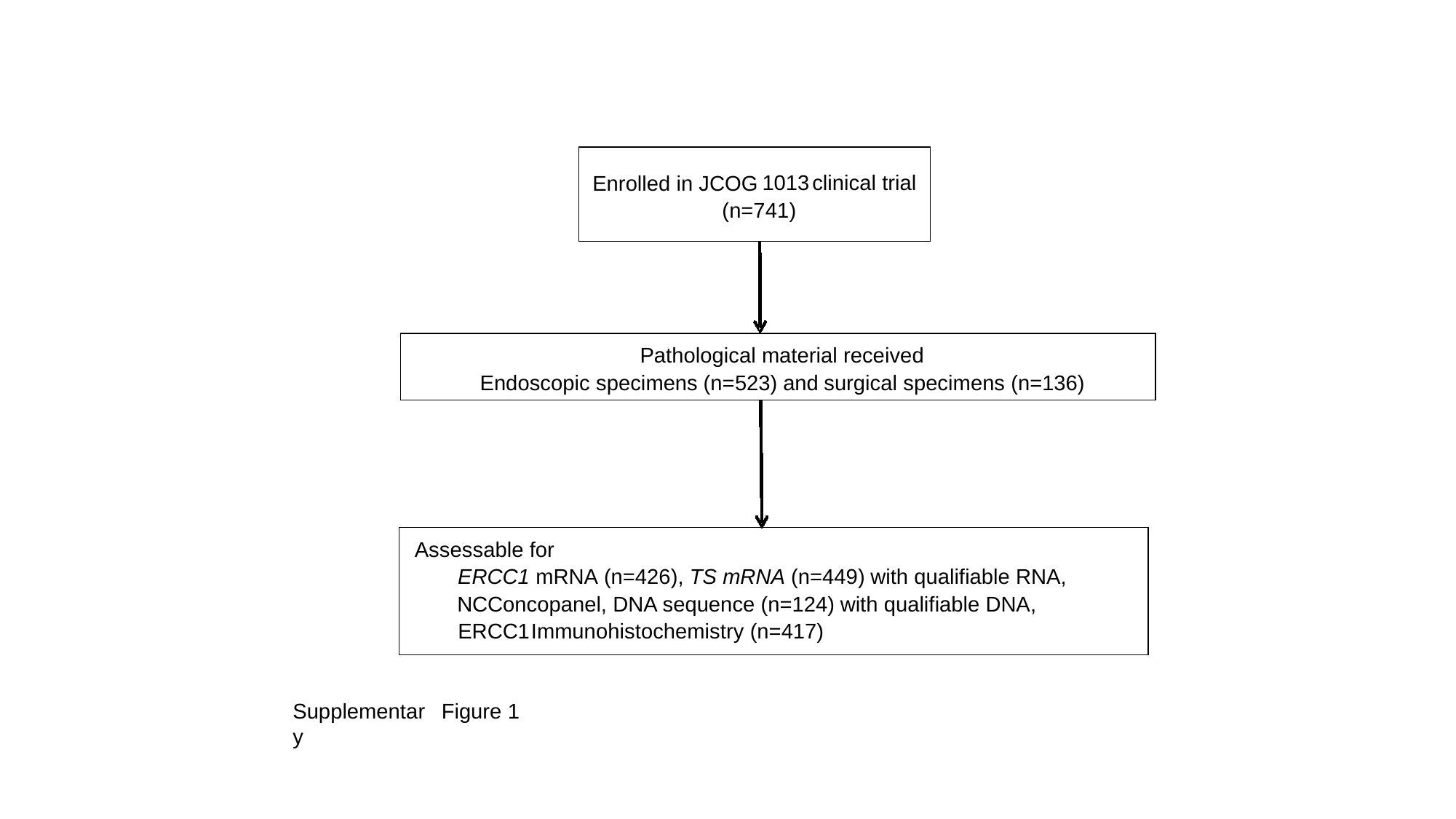

1013
clinical trial
Enrolled in JCOG
(n=7
41
)
Pathological material received
Endoscopic specimens
(n=
523
)
and
surgical specimens (n=136)
Assessable for
ERCC1
mRNA
(n=
426
),
TS
mRNA
(n=
449
)
with qualifiable RNA,
NCConcopanel, DNA sequence (n=124)
with qualifiable DNA,
ERCC
1
Immunohistochemistry (n=417)
Supplementary
Figure 1

## Slide 2
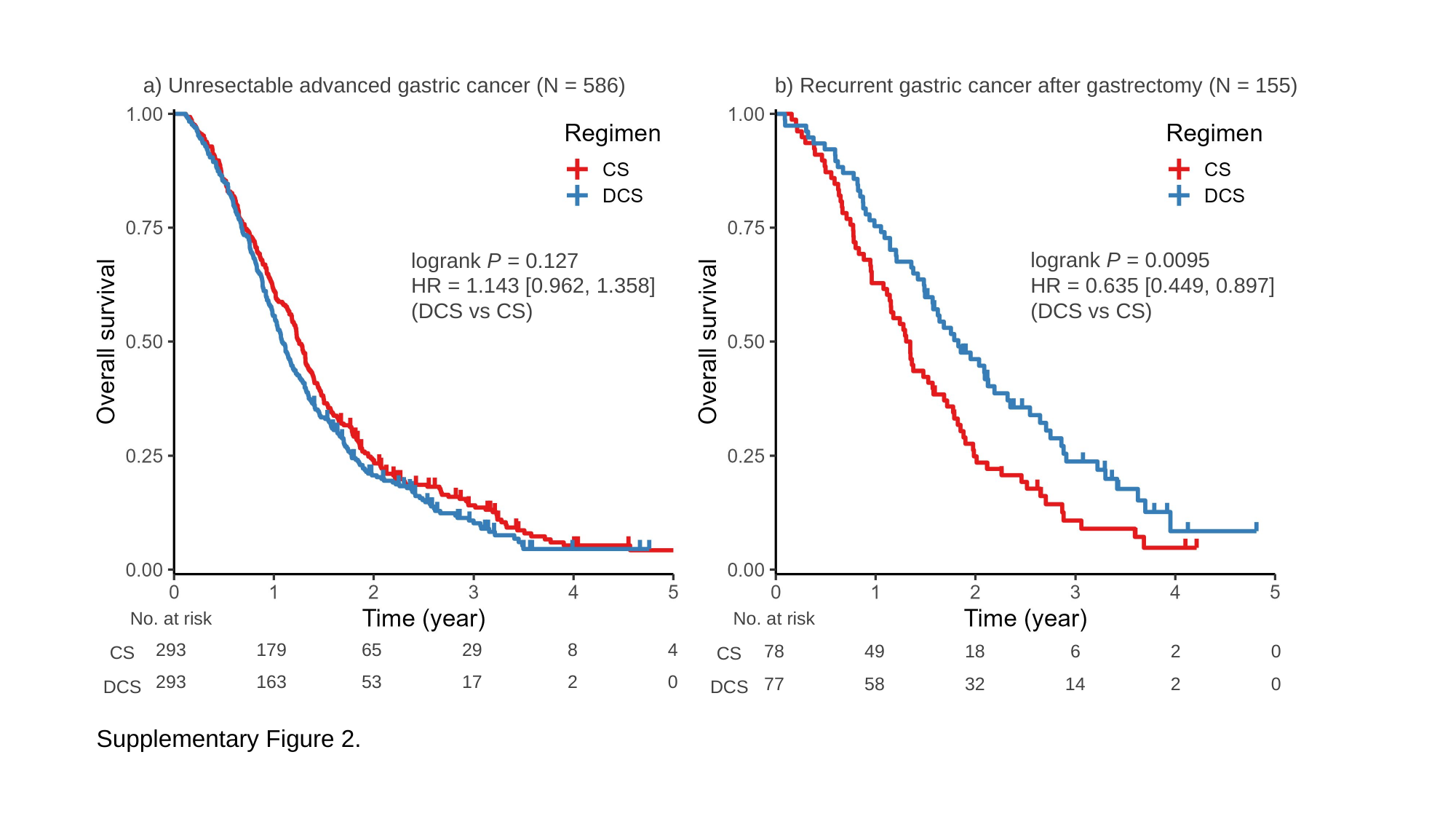

a) Unresectable advanced gastric cancer (N = 586)
b) Recurrent gastric cancer after gastrectomy (N = 155)
logrank P = 0.0095
HR = 0.635 [0.449, 0.897]
(DCS vs CS)
logrank P = 0.127
HR = 1.143 [0.962, 1.358]
(DCS vs CS)
| No. at risk | | | | | |
| --- | --- | --- | --- | --- | --- |
| 293 | 179 | 65 | 29 | 8 | 4 |
| 293 | 163 | 53 | 17 | 2 | 0 |
| No. at risk | | | | | |
| --- | --- | --- | --- | --- | --- |
| 78 | 49 | 18 | 6 | 2 | 0 |
| 77 | 58 | 32 | 14 | 2 | 0 |
CS
CS
DCS
DCS
# Supplementary Figure 2.
