# Supplementary material for: Predictive and prognostic value of excision repair cross-complementing group 1 in patients with advanced gastric cancer
Source: BJC Rep. 2024 Mar 5;2:18. doi: 10.1038/s44276-024-00046-w (PMC11523942; doi:10.1038/s44276-024-00046-w)
Supplement: Supplementary file 3 — Supplementary table [file 44276_2024_46_MOESM3_ESM.docx]

Supplementary Table 1. Detail of the genes added to the modified NCC Oncopanel

| Chr | REF | ALT | *GENE* | Func.refGeneWithVer | Gene.refGeneWithVer | ExonicFunc.refGeneWithVer | avsnp150 |
| --- | --- | --- | --- | --- | --- | --- | --- |
| chr1 | G | T | *DPYD_1* | exonic | *DPYD* | nonsynonymous SNV | rs56005131 |
| chr1 | C | T | *DPYD_2* | exonic | *DPYD* | nonsynonymous SNV | rs1801160 |
| chr1 | A | G | *DPYD_3* | exonic | *DPYD* | synonymous SNV | rs17376848 |
| chr1 | T | C | *DPYD_4* | exonic | *DPYD* | nonsynonymous SNV | rs1801159 |
| chr1 | C | T | *DPYD_5* | exonic | *DPYD* | nonsynonymous SNV | rs148994843 |
| chr1 | C | . | *NA_SNP_1* | NA | *NA* | NA | NA |
| chr1 | T | C | *DPYD_6* | exonic | *DPYD* | nonsynonymous SNV | rs2297595 |
| chr1 | G | A | *DPYD_7* | exonic | *DPYD* | nonsynonymous SNV | rs1801265 |
| chr6 | T | C | *ATXN1* | intronic | *ATXN1* | NA | rs179980 |
| chr6 | A | C | *MRPS18A_VEGFA_1* | intergenic | *MRPS18A;VEGFA* | NA | rs699947 |
| chr6 | GGTCCC | . | *MRPS18A_VEGFA_2* | intergenic | *MRPS18A;VEGFA* | NA | NA |
| chr6 | G | . | *NA_SNP_2* | NA | *NA* | NA | NA |
| chr6 | C | T | *VEGFA_1* | upstream | *VEGFA* | NA | rs833061 |
| chr6 | A | G | *VEGFA_2* | upstream | *VEGFA* | NA | rs1570360 |
| chr6 | C | G | *VEGFA_3* | UTR5 | *VEGFA* | NA | rs2010963 |
| chr6 | C | T | *VEGFA_4* | exonic | *VEGFA* | synonymous SNV | rs25648 |
| chr6 | C | T | *VEGFA_5* | intronic | *VEGFA* | NA | rs3025000 |
| chr6 | C | T | *VEGFA_6* | UTR3 | *VEGFA* | NA | rs3025039 |
| chr6 | C | T | *VEGFA_7* | UTR3 | *VEGFA* | NA | rs3025040 |
| chr6 | C | T | *VEGFA_LINC02537_1* | intergenic | *VEGFA;LINC02537* | NA | rs6900017 |
| chr6 | T | C | *VEGFA_LINC02537_2* | intergenic | *VEGFA;LINC02537* | NA | rs9369421 |
| chr6 | A | G | *VEGFA_LINC02537_3* | intergenic | *VEGFA;LINC02537* | NA | rs879825 |
| chr7 | T | C | *ABCB1_1* | UTR3 | *ABCB1* | NA | rs3842 |
| chr7 | A | C | *ABCB1_2* | intronic | *ABCB1* | NA | rs2235047 |
| chr7 | A | G | *ABCB1_3* | exonic | *ABCB1* | synonymous SNV | rs1045642 |
| chr7 | C | T | *ABCB1_4* | intronic | *ABCB1* | NA | rs28401781 |
| chr7 | C | T | *ABCB1_5* | intronic | *ABCB1* | NA | rs2235067 |
| chr7 | A | G | *ABCB1_6* | intronic | *ABCB1* | NA | rs4148740 |
| chr7 | A | C | *ABCB1_7* | intronic | *ABCB1* | NA | rs10280101 |
| chr7 | G | A | *ABCB1_8* | intronic | *ABCB1* | NA | rs7787082 |
| chr7 | A | G | *ABCB1_9* | intronic | *ABCB1* | NA | rs2032583 |
| chr7 | A | C,T | *ABCB1_10* | exonic | *ABCB1* | nonsynonymous SNV | rs2032582 |
| chr7 | T | C | *ABCB1_11* | intronic | *ABCB1* | NA | rs4148739 |
| chr7 | T | C | *ABCB1_12* | intronic | *ABCB1* | NA | rs11983225 |
| chr7 | C | T | *ABCB1_13* | intronic | *ABCB1* | NA | rs4148738 |
| chr7 | A | G | *ABCB1_14* | intronic | *ABCB1* | NA | rs10248420 |
| chr7 | C | T | *ABCB1_15* | intronic | *ABCB1* | NA | rs2235040 |
| chr7 | C | T | *ABCB1_16* | intronic | *ABCB1* | NA | rs12720067 |
| chr7 | T | C | *ABCB1_17* | intronic | *ABCB1* | NA | rs4148737 |
| chr7 | A | T | *ABCB1_18* | intronic | *ABCB1* | NA | rs1922242 |
| chr7 | A | G | *ABCB1_19* | exonic | *ABCB1* | synonymous SNV | rs1128503 |
| chr7 | C | A | *ABCB1_20* | intronic | *ABCB1* | NA | rs2235015 |
| chr7 | A | G | *ABCB1_21* | intronic | *ABCB1* | NA | rs3789243 |
| chr7 | A | G | *ABCB1_22* | UTR5 | *ABCB1* | NA | rs3213619 |
| chr7 | G | A | *ABCB1_23* | intronic | *ABCB1* | NA | rs4728709 |
| chr7 | G | A | *ABCB1_RUNDC3B_1* | intronic | *ABCB1;RUNDC3B* | NA | rs10267099 |
| chr7 | T | C | *RUNDC3B_1* | intronic | *RUNDC3B* | NA | rs28656907 |
| chr7 | G | T | *RUNDC3B_2* | intronic | *RUNDC3B* | NA | rs17160359 |
| chr8 | T | G | *PRKDC_1* | intronic | *PRKDC* | NA | rs8178085 |
| chr8 | G | A | *PRKDC_2* | intronic | *PRKDC* | NA | rs12334811 |
| chr10 | G | A | *MGMT* | intronic | *MGMT* | NA | rs4751104 |
| chr11 | A | G | *GSTP1* | exonic | *GSTP1* | nonsynonymous SNV | rs1695 |
| chr11 | G | T | *ACRV1* | upstream | *ACRV1* | NA | rs11220184 |
| chr18 | T | C | *TYMSOS* | intronic | *TYMSOS* | NA | rs2853741 |
| chr18 | C | . | *NA_SNP_3* | NA | *NA* | NA | NA |
| chr18 | G | A | *TYMS* | intronic | *TYMS* | NA | rs2847153 |
| chr19 | T | C | *XRCC1_1* | exonic | *XRCC1* | nonsynonymous SNV | rs25487 |
| chr19 | G | A | *XRCC1_2* | exonic | *XRCC1* | nonsynonymous SNV | rs1799782 |
| chr19 | A | C | *POLR1G_1* | exonic | *POLR1G* | nonsynonymous SNV | rs735482 |
| chr19 | G | A | *POLR1G_2* | exonic | *POLR1G* | nonsynonymous SNV | rs2336219 |
| chr19 | C | A | *POLR1G_3* | exonic | *POLR1G* | nonsynonymous SNV | rs3212986 |
| chr19 | T | G | *ERCC1_1* | UTR3 | *ERCC1* | NA | rs3212980 |
| chr19 | C | T | *ERCC1_2* | intronic | *ERCC1* | NA | rs3212964 |
| chr19 | A | G | *ERCC1_3* | exonic | *ERCC1* | synonymous SNV | rs11615 |
| chr19 | G | C | *ERCC1_4* | intronic | *ERCC1* | NA | rs3212948 |
| chr19 | C | A | *ERCC1_5* | intronic | *ERCC1* | NA | rs2298881 |

Chr, chromosome; ref, reference; ALT, alteration

Supplementary Table 2. The incidence of single nucleotide polymorphisms in the genes added to NCC Oncopanel

| **Variable** | **N** | **CS**, N = 372^1^ | **DCS**, N = 370^1^ | **p-value**^2^ |
| --- | --- | --- | --- | --- |
| DPYD_1 | 124 |  |  | >0.9 |
| GG |  | 57 (93%) | 58 (92%) |  |
| GT |  | 4 (6.6%) | 5 (7.9%) |  |
| DPYD_2 | 124 |  |  | >0.9 |
| CC |  | 58 (95%) | 60 (95%) |  |
| CT |  | 3 (4.9%) | 3 (4.8%) |  |
| DPYD_3 | 124 |  |  | 0.7 |
| AA |  | 40 (66%) | 46 (73%) |  |
| AG |  | 20 (33%) | 16 (25%) |  |
| GG |  | 1 (1.6%) | 1 (1.6%) |  |
| DPYD_4 | 124 |  |  | 0.092 |
| CC |  | 6 (9.8%) | 4 (6.3%) |  |
| TC |  | 31 (51%) | 22 (35%) |  |
| TT |  | 24 (39%) | 37 (59%) |  |
| DPYD_5 | 124 |  |  | 0.5 |
| CC |  | 60 (98%) | 63 (100%) |  |
| CT |  | 1 (1.6%) | 0 (0%) |  |
| DPYD_6 | 124 |  |  | 0.8 |
| TC |  | 5 (8.2%) | 6 (9.5%) |  |
| TT |  | 56 (92%) | 57 (90%) |  |
| DPYD_7 | 124 |  |  | >0.9 |
| AA |  | 55 (90%) | 57 (90%) |  |
| GA |  | 6 (9.8%) | 6 (9.5%) |  |
| MRPS18A_VEGFA_1 | 124 |  |  | 0.7 |
| AA |  | 8 (13%) | 11 (17%) |  |
| AC |  | 23 (38%) | 25 (40%) |  |
| CC |  | 30 (49%) | 27 (43%) |  |
| VEGFA_1 | 124 |  |  | 0.7 |
| CC |  | 8 (13%) | 11 (17%) |  |
| CT |  | 23 (38%) | 25 (40%) |  |
| TT |  | 30 (49%) | 27 (43%) |  |
| VEGFA_2 | 124 |  |  | >0.9 |
| AA |  | 1 (1.6%) | 2 (3.2%) |  |
| AG |  | 18 (30%) | 19 (30%) |  |
| GG |  | 42 (69%) | 42 (67%) |  |
| VEGFA_3 | 124 |  |  | 0.14 |
| CC |  | 12 (20%) | 11 (17%) |  |
| CG |  | 33 (54%) | 25 (40%) |  |
| GG |  | 16 (26%) | 27 (43%) |  |
| VEGFA_4 | 124 |  |  | 0.3 |
| CC |  | 43 (70%) | 44 (70%) |  |
| CT |  | 17 (28%) | 14 (22%) |  |
| TT |  | 1 (1.6%) | 5 (7.9%) |  |
| VEGFA_5 | 124 |  |  | 0.10 |
| CC |  | 16 (26%) | 28 (44%) |  |
| CT |  | 33 (54%) | 24 (38%) |  |
| TT |  | 12 (20%) | 11 (17%) |  |
| VEGFA_6 | 124 |  |  | >0.9 |
| CC |  | 36 (59%) | 39 (62%) |  |
| CT |  | 23 (38%) | 22 (35%) |  |
| TT |  | 2 (3.3%) | 2 (3.2%) |  |
| VEGFA_7 | 124 |  |  | 0.9 |
| CC |  | 39 (64%) | 38 (60%) |  |
| CT |  | 20 (33%) | 23 (37%) |  |
| TT |  | 2 (3.3%) | 2 (3.2%) |  |
| VEGFA_LINC02537_1 | 124 |  |  | 0.8 |
| CC |  | 36 (59%) | 41 (65%) |  |
| CT |  | 21 (34%) | 18 (29%) |  |
| TT |  | 4 (6.6%) | 4 (6.3%) |  |
| VEGFA_LINC02537_2 | 124 |  |  | >0.9 |
| CC |  | 3 (4.9%) | 3 (4.8%) |  |
| TC |  | 23 (38%) | 21 (33%) |  |
| TT |  | 35 (57%) | 39 (62%) |  |
| VEGFA_LINC02537_3 | 124 |  |  | 0.4 |
| AA |  | 35 (57%) | 39 (62%) |  |
| AG |  | 23 (38%) | 18 (29%) |  |
| GG |  | 3 (4.9%) | 6 (9.5%) |  |
| ABCB1_1 | 124 |  |  | >0.9 |
| CC |  | 2 (3.3%) | 3 (4.8%) |  |
| TC |  | 28 (46%) | 27 (43%) |  |
| TT |  | 31 (51%) | 33 (52%) |  |
| ABCB1_2 | 124 |  |  | 0.3 |
| AA |  | 18 (30%) | 26 (41%) |  |
| AC |  | 36 (59%) | 28 (44%) |  |
| CC |  | 7 (11%) | 9 (14%) |  |
| ABCB1_3 | 124 |  |  | 0.8 |
| AA |  | 9 (15%) | 12 (19%) |  |
| AG |  | 31 (51%) | 29 (46%) |  |
| GG |  | 21 (34%) | 22 (35%) |  |
| ABCB1_4 | 124 |  |  | 0.8 |
| CC |  | 56 (92%) | 55 (87%) |  |
| CT |  | 4 (6.6%) | 7 (11%) |  |
| TT |  | 1 (1.6%) | 1 (1.6%) |  |
| ABCB1_5 | 124 |  |  | 0.4 |
| CC |  | 56 (92%) | 53 (84%) |  |
| CT |  | 4 (6.6%) | 9 (14%) |  |
| TT |  | 1 (1.6%) | 1 (1.6%) |  |
| ABCB1_6 | 123 |  |  | 0.8 |
| AA |  | 56 (92%) | 54 (87%) |  |
| AG |  | 4 (6.6%) | 7 (11%) |  |
| GG |  | 1 (1.6%) | 1 (1.6%) |  |
| ABCB1_7 | 124 |  |  | 0.8 |
| AA |  | 56 (92%) | 55 (87%) |  |
| AC |  | 4 (6.6%) | 7 (11%) |  |
| CC |  | 1 (1.6%) | 1 (1.6%) |  |
| ABCB1_8 | 124 |  |  | 0.2 |
| AA |  | 7 (11%) | 9 (14%) |  |
| GA |  | 37 (61%) | 28 (44%) |  |
| GG |  | 17 (28%) | 26 (41%) |  |
| ABCB1_9 | 124 |  |  | 0.8 |
| AA |  | 56 (92%) | 55 (87%) |  |
| AG |  | 4 (6.6%) | 7 (11%) |  |
| GG |  | 1 (1.6%) | 1 (1.6%) |  |
| ABCB1_10 | 85 |  |  | 0.3 |
| AA |  | 5 (12%) | 10 (23%) |  |
| AC or AT |  | 26 (63%) | 21 (48%) |  |
| CC or TT |  | 10 (24%) | 13 (30%) |  |
| ABCB1_11 | 124 |  |  | 0.8 |
| CC |  | 1 (1.6%) | 1 (1.6%) |  |
| TC |  | 4 (6.6%) | 7 (11%) |  |
| TT |  | 56 (92%) | 55 (87%) |  |
| ABCB1_12 | 124 |  |  | 0.4 |
| CC |  | 1 (1.6%) | 0 (0%) |  |
| TC |  | 4 (6.6%) | 8 (13%) |  |
| TT |  | 56 (92%) | 55 (87%) |  |
| ABCB1_13 | 124 |  |  | 0.5 |
| CC |  | 6 (9.8%) | 10 (16%) |  |
| CT |  | 36 (59%) | 31 (49%) |  |
| TT |  | 19 (31%) | 22 (35%) |  |
| ABCB1_14 | 124 |  |  | 0.042 |
| AA |  | 16 (26%) | 27 (43%) |  |
| AG |  | 38 (62%) | 25 (40%) |  |
| GG |  | 7 (11%) | 11 (17%) |  |
| ABCB1_15 | 124 |  |  | 0.8 |
| CC |  | 56 (92%) | 55 (87%) |  |
| CT |  | 4 (6.6%) | 7 (11%) |  |
| TT |  | 1 (1.6%) | 1 (1.6%) |  |
| ABCB1_16 | 124 |  |  | 0.8 |
| CC |  | 56 (92%) | 55 (87%) |  |
| CT |  | 4 (6.6%) | 7 (11%) |  |
| TT |  | 1 (1.6%) | 1 (1.6%) |  |
| ABCB1_17 | 124 |  |  | 0.9 |
| CC |  | 8 (13%) | 8 (13%) |  |
| TC |  | 28 (46%) | 32 (51%) |  |
| TT |  | 25 (41%) | 23 (37%) |  |
| ABCB1_18 | 124 |  |  | >0.9 |
| AA |  | 24 (39%) | 23 (37%) |  |
| AT |  | 29 (48%) | 32 (51%) |  |
| TT |  | 8 (13%) | 8 (13%) |  |
| ABCB1_19 | 124 |  |  | 0.8 |
| AA |  | 21 (34%) | 19 (30%) |  |
| AG |  | 31 (51%) | 32 (51%) |  |
| GG |  | 9 (15%) | 12 (19%) |  |
| ABCB1_20 | 124 |  |  | 0.6 |
| AA |  | 1 (1.6%) | 1 (1.6%) |  |
| CA |  | 3 (4.9%) | 7 (11%) |  |
| CC |  | 57 (93%) | 55 (87%) |  |
| ABCB1_21 | 124 |  |  | 0.8 |
| AA |  | 7 (11%) | 6 (9.5%) |  |
| AG |  | 25 (41%) | 30 (48%) |  |
| GG |  | 29 (48%) | 27 (43%) |  |
| ABCB1_22 | 124 |  |  | 0.076 |
| AA |  | 57 (93%) | 53 (84%) |  |
| AG |  | 3 (4.9%) | 10 (16%) |  |
| GG |  | 1 (1.6%) | 0 (0%) |  |
| ABCB1_23 | 124 |  |  | >0.9 |
| AA |  | 2 (3.3%) | 2 (3.2%) |  |
| GA |  | 15 (25%) | 17 (27%) |  |
| GG |  | 44 (72%) | 44 (70%) |  |
| ABCB1_RUNDC3B_1 | 124 |  |  | 0.6 |
| AA |  | 59 (97%) | 60 (95%) |  |
| GA |  | 1 (1.6%) | 3 (4.8%) |  |
| GG |  | 1 (1.6%) | 0 (0%) |  |
| RUNDC3B_1 | 124 |  |  | 0.8 |
| CC |  | 16 (26%) | 14 (22%) |  |
| TC |  | 29 (48%) | 34 (54%) |  |
| TT |  | 16 (26%) | 15 (24%) |  |
| RUNDC3B_2 | 124 |  |  | 0.4 |
| GG |  | 56 (92%) | 55 (87%) |  |
| GT |  | 5 (8.2%) | 8 (13%) |  |
| PRKDC_1 | 124 |  |  | 0.3 |
| TG |  | 8 (13%) | 5 (7.9%) |  |
| TT |  | 53 (87%) | 58 (92%) |  |
| PRKDC_2 | 124 |  |  | 0.059 |
| AA |  | 5 (8.2%) | 0 (0%) |  |
| GA |  | 16 (26%) | 15 (24%) |  |
| GG |  | 40 (66%) | 48 (76%) |  |
| MGMT | 124 |  |  | 0.6 |
| AA |  | 1 (1.6%) | 1 (1.6%) |  |
| GA |  | 10 (16%) | 6 (9.5%) |  |
| GG |  | 50 (82%) | 56 (89%) |  |
| GSTP1 | 124 |  |  | 0.2 |
| AA |  | 41 (67%) | 44 (70%) |  |
| AG |  | 15 (25%) | 18 (29%) |  |
| GG |  | 5 (8.2%) | 1 (1.6%) |  |
| ACRV1 | 124 |  |  | 0.8 |
| GG |  | 38 (62%) | 41 (65%) |  |
| GT |  | 21 (34%) | 21 (33%) |  |
| TT |  | 2 (3.3%) | 1 (1.6%) |  |
| TYMSOS | 124 |  |  | 0.8 |
| CC |  | 16 (26%) | 18 (29%) |  |
| TC |  | 30 (49%) | 33 (52%) |  |
| TT |  | 15 (25%) | 12 (19%) |  |
| TYMS | 124 |  |  | >0.9 |
| AA |  | 9 (15%) | 10 (16%) |  |
| GA |  | 31 (51%) | 33 (52%) |  |
| GG |  | 21 (34%) | 20 (32%) |  |
| XRCC1_1 | 124 |  |  | 0.4 |
| CC |  | 30 (49%) | 36 (57%) |  |
| TC |  | 26 (43%) | 25 (40%) |  |
| TT |  | 5 (8.2%) | 2 (3.2%) |  |
| XRCC1_2 | 124 |  |  | 0.6 |
| AA |  | 9 (15%) | 6 (9.5%) |  |
| GA |  | 27 (44%) | 28 (44%) |  |
| GG |  | 25 (41%) | 29 (46%) |  |
| POLR1G_1 | 124 |  |  | 0.2 |
| AA |  | 12 (20%) | 19 (30%) |  |
| AC |  | 28 (46%) | 31 (49%) |  |
| CC |  | 21 (34%) | 13 (21%) |  |
| POLR1G_2 | 124 |  |  | 0.2 |
| AA |  | 21 (34%) | 13 (21%) |  |
| GA |  | 28 (46%) | 31 (49%) |  |
| GG |  | 12 (20%) | 19 (30%) |  |
| POLR1G_3 | 124 |  |  | 0.2 |
| AA |  | 3 (4.9%) | 4 (6.3%) |  |
| CA |  | 13 (21%) | 22 (35%) |  |
| CC |  | 45 (74%) | 37 (59%) |  |
| ERCC1_1 | 124 |  |  | 0.2 |
| GG |  | 3 (4.9%) | 3 (4.8%) |  |
| TG |  | 13 (21%) | 23 (37%) |  |
| TT |  | 45 (74%) | 37 (59%) |  |
| ERCC1_2 | 124 |  |  | 0.5 |
| CC |  | 13 (21%) | 16 (25%) |  |
| CT |  | 30 (49%) | 34 (54%) |  |
| TT |  | 18 (30%) | 13 (21%) |  |
| ERCC1_3 | 124 |  |  | 0.4 |
| AA |  | 4 (6.6%) | 7 (11%) |  |
| AG |  | 27 (44%) | 21 (33%) |  |
| GG |  | 30 (49%) | 35 (56%) |  |
| ERCC1_4 | 124 |  |  | 0.5 |
| CC |  | 30 (49%) | 34 (54%) |  |
| GC |  | 27 (44%) | 22 (35%) |  |
| GG |  | 4 (6.6%) | 7 (11%) |  |
| ERCC1_5 | 124 |  |  | 0.4 |
| AA |  | 17 (28%) | 12 (19%) |  |
| CA |  | 28 (46%) | 29 (46%) |  |
| CC |  | 16 (26%) | 22 (35%) |  |
| ^1^n (%) | | | | |
| ^2^Fisher's exact test; Pearson's Chi-squared test | | | | |
